# Iloprost in pulmonary hypertension due to sub-massive pulmonary embolism: report of two cases

**DOI:** 10.3402/ljm.v8i0.22391

**Published:** 2013-08-16

**Authors:** Abdullah Hassan Alsaghir, Soror Abdullah Alaithan, Basima Alsihati, Dhia Naji Alhajjaj

**Affiliations:** Department of Medicine, Dammam Medical Complex, Dammam, Saudi Arabia

Patients with acute pulmonary embolism (PE) who has normal systemic arterial pressure and right ventricular (RV) dysfunction are considered to have sub-massive pulmonary embolism (SPE), with 3-month mortality risk of 21% (1). The benefit of the current therapy other than anticoagulation including thrombolytic therapy (TT), embolectomy, and IVC filter insertion, in the absence of cardiogenic shock, is still debated (2). Vasoconstrictive mediators (including thromboxane-A and serotonin) have an important role to play in the increase of peripheral vascular resistance (PVR) and ultimately in developing pulmonary hypertension (PH) in acute phase (3, 4). A study by Idrees et al. described a beneficial effect of inhaled iloprost in five patients with SPE, in whom there is an improvement in WHO-functional class (WHO-FC), 6-min walk distance (6MWD) test, and echocardiographic parameters (5). Iloprost, an inhaled prostacyclin approved for the treatment of patients with pulmonary arterial hypertension, exerts long-term benefits through antiproliferative and antithrombotic effects, but is also a potent acute pulmonary vasodilator with the duration of action of approximately 60 min (6).

Two patients were evaluated at the Dammam Medical Complex, Dammam, Saudi Arabia between June and December 2012. The diagnosis of PE was established by helical computed tomography (CT) angiogram that showed a thrombus at both pulmonary arteries at presentation. They had normal systemic blood pressure, PH documented by echocardiography demonstrated an estimated systolic pulmonary artery pressure >45 mm Hg and findings of RV dysfunction suggestive of acute changes. Both patients received therapeutic anticoagulation with low-molecular-weight heparin and targeted warfarin dose to international normalized ratio of 2–3. They were still symptomatic and required more oxygen, for which they were offered TT. They declined TT, but agreed to receive inhaled iloprost through a nebulized device of 2.5–5.0 µg per inhalation for 15 min, 8× daily for 3 months.

**Fig. 1:**
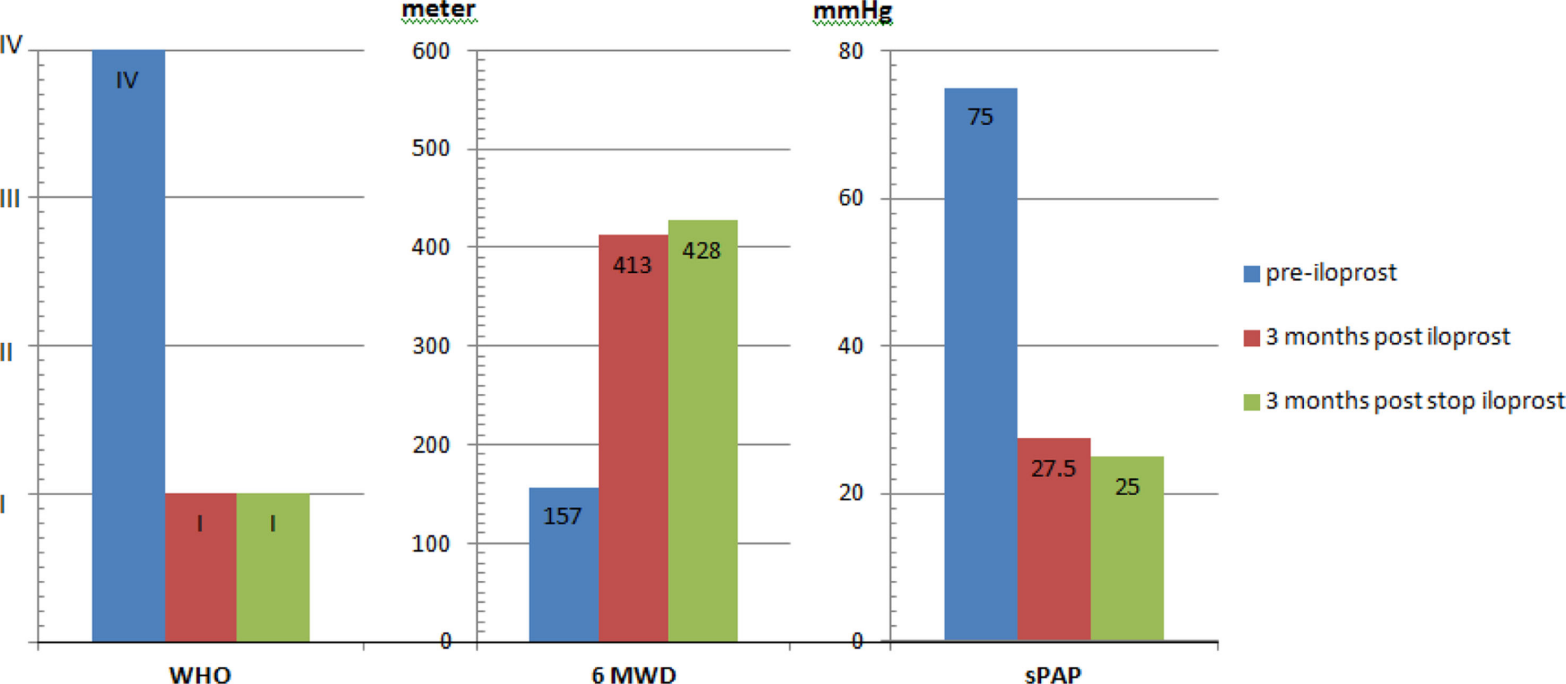
The mean of WHO functional class, 6MWD, and sPAP (by echo) pre- and 3 months post and 3 months post-cessation of iloprost.

The main efficacy index was defined as the changes in WHO-FC, 6MWD, oxygen saturation, and echocardiography parameters during 3-month inhalation therapy. These parameters were assessed at baseline, 3-month post-inhalation, and 3-month post-cessation of iloprost. Both patients tolerated the treatment well. They showed significant improvement in their clinical symptoms and echocardiographic parameters (Table 1, Figure 1).

**Table 1:** Clinical and physiological parameters of both patients before and after iloprost (3 months before and 3 months post-cessation of iloprost)

Measurement	Pre-iloprost	3 months post-iloprost	3 months post-cessation of iloprost
**Case 1**			
WHO functional class	III	I	I
6MWD	176 m	408 m	422 m
Oxygen saturation	90% on RA	97% on RA	97% on RA
	98% on 2 L/min		
sPAP (by echo)	>70 mm Hg	30 mm Hg	25 mm Hg
RV dysfunction	Severe	Normal	Normal
Qualitative score			
**Case 2**			
WHO functional class	IV	I	I
6MWD	138 m	418 m	434 m
Oxygen saturation	89% on RA	98% on RA	98% on RA
	97% on 2 L/min		
sPAP (by echo)	80 mm Hg	25 mm Hg	25 mm Hg
RV dysfunction	Moderate	Normal	Normal
Qualitative score			

In conclusion, iloprost might have favorable acute effects on symptoms, exercise tolerance, and pulmonary hypertension in sub-massive pulmonary embolism.

*Abdullah Hassan Alsaghir* Department of Medicine Dammam Medical Complex Dammam, Saudi Arabia Email: asaghir4000@yahoo.com *Soror Abdullah Alaithan* Department of Medicine Dammam Medical Complex Dammam, Saudi Arabia *Basima Alsihati* Department of Medicine Dammam Medical Complex Dammam, Saudi Arabia *Dhia Naji Alhajjaj* Department of Medicine Dammam Medical Complex Dammam, Saudi Arabia
